# Functional and Bioactive Properties of Wheat Protein Fractions: Impact of Digestive Enzymes on Antioxidant, α-Amylase, and Angiotensin-Converting Enzyme Inhibition Potential

**DOI:** 10.3390/molecules28166012

**Published:** 2023-08-11

**Authors:** Sana Gammoh, Muhammad H. Alu’datt, Mohammad N. Alhamad, Carole C. Tranchant, Taha Rababah, Doa’a Al-U’datt, Neveen Hussein, Mohammad Alrosan, Thuan-Chew Tan, Stan Kubow, Haya Alzoubi, Ali Almajwal

**Affiliations:** 1 Department of Nutrition and Food Technology, Faculty of Agriculture, Jordan University of Science and Technology, Irbid 22110, Jordan; trababah@just.edu.jo (T.R.); neveenoo91@yahoo.com (N.H.); haia_zoubi@yahoo.com (H.A.); 2 Department of Natural Resources and Environment, Faculty of Agriculture, Jordan University of Science and Technology, Irbid 22110, Jordan; malhamad@just.edu.jo; 3 School of Food Science, Nutrition and Family Studies, Faculty of Health Sciences and Community Services, Université de Moncton, Moncton, NB E1A 3E9, Canada; 4 Department of Physiology and Biochemistry, Faculty of Medicine, Jordan University of Science and Technology, Irbid 22110, Jordan; dgaludatt@just.edu.jo; 5 Applied Science Research Center, Applied Science Private University, Amman 11937, Jordan; mohammad.alrosan@hotmail.com; 6 Food Technology Division, School of Industrial Technology, Universiti Sains Malaysia, Penang 11800, Malaysia; thuanchew@usm.my; 7 School of Dietetics and Human Nutrition, McGill University, Montreal, QC H9X 3V9, Canada; stankubow@gmail.com; 8 Department of Community Health Sciences, College of Applied Medical Sciences, King Saud University, Riyadh 12372, Saudi Arabia; aalmajwal@ksu.edu.sa

**Keywords:** wheat proteins, biological activity, enzymatic hydrolysates, endopeptidases, antioxidant activity, angiotensin-I converting enzyme inhibition, α-amylase inhibition

## Abstract

This research aimed to determine the biofunctional properties of wheat flour (WF) protein fractions and modifications to the antioxidant, anti-α-amylase and anti-angiotensin-I converting enzyme (ACE) activities induced by the action of digestive endopeptidases in vitro. A molecular characterization of the most abundant protein fractions, i.e., albumins, glutelins-1, glutelins-2 and prolamins, showed that low- and high-MW polypeptides rich in cysteine, glutamic acid and leucine were present in albumins and glutelins, whereas low-MW subunits with a high proportion of polar amino acids prevailed in prolamins. Prolamins exhibited the second-highest water holding capacity (54%) after WF (84%), while albumins provided superior foam stability (76%). Prolamins, glutenins-1 and globulins demonstrated the highest antioxidant activity (up to 95%, 68% and 59%, respectively) both before and after hydrolysis with pepsin (P-H) or trypsin–chymotrypsin (TC-H). Prolamins, globulins and WF strongly inhibited α-amylase (>90%) before and after TC-H, and before P-H (55–71%). Moreover, P-H significantly increased α-amylase inhibition by albumins from 53 to 74%. The fractions with strong ACE inhibitory activity (70–89%) included prolamins and globulins after TC-H or P-H, as well as globulins before TC-H and WF before P-H. This novel evidence indicates that WF protein fractions and their peptide-enriched P and TC hydrolysates are excellent sources of multifunctional bioactives with antioxidant, antihyperglycemic and antihypertensive potential.

## 1. Introduction

Wheat plays a prominent role in ensuring global food and nutrition security. Not only does this crop supply about one-fifth of the world’s dietary calories and protein [1], it also provides essential micronutrients and a variety of other health-promoting bioactive phytochemicals, including phenolic compounds and alkylresorcinols [2]. The modern wheat varieties, bread wheat (*Triticum aestivum* L.) and durum or pasta wheat (*Triticum durum* Desf.), account for *ca*. 95% and 5% of the global production of wheat [3]. The long-lasting legacy and versatility of this cereal are closely associated with the capacity of its gluten proteins, which make up to 80% of the endosperm proteins, to form viscoelastic doughs that enable the transformation of wheat flour (WF) into a wide range of relatively inexpensive staple foods, notably breads, noodles, pastries and snack foods, to name a few [3]. Gluten’s high elasticity and extensibility is a major determinant of WF technological functionality [4]. Moreover, a growing number of functional foods [5] and pharmaceutical applications (e.g., drug delivery nanocarriers) [6] have been proposed recently, by attempting to deliver less well-understood functionalities and health effects from wheat components.

Wheat grain proteins have traditionally been classified into four so-called Osborne fractions, according to their solubility in water (albumins), salt solutions (globulins), aqueous alcohols (gliadins), or dilute acid or alkaline solutions (glutenins) [7]. This classification offers a practical means by which to recover gluten proteins, namely gliadins and glutenins, as well as nongluten proteins, mainly albumins and globulins, which contain regulatory and protective proteins, such as the enzymes and enzyme inhibitors involved in the metabolic activity of seeds [8]. Wheat gliadins (prolamins) are further subdivided into α-, γ-, ω1,2- and ω5-gliadins based on their molecular weight (25–75 kDa) [8,9]. Prolamins are predominantly monomeric in their native state, whereas glutenins (glutelins) occur as polymers identified as low- (30–50 kDa) and high-molecular weight (70–140 kDa) subunits [9]. WF protein fractions represent a rich pool of substrates that could be utilized to yield ingredients with enhanced or novel biological and functional activities, but there are limited data on their health-promoting effects. Previous reports in this area were mainly focused on wheat non-nutrient compounds [2], wheat gluten [10,11] or unfractionated wheat germ proteins [12,13]. The presence of phenolic compounds in WF, including high levels of phenolic acids [14,15], is noteworthy, as these compounds can interact with proteins through covalent and noncovalent binding [16], which could confer unique or modified functionalities to the conjugated proteins. Antioxidant action and the inhibition of target metabolic enzymes associated with chronic diseases, such as the angiotensin-I converting enzyme (ACE) in hypertension prevention/management and α-amylase in glycemic control and weight management [17,18], are among the potential pharmacological uses of phenolic compounds.

Peptides are another class of biomolecules that can exert protective health effects. One of the safest and most economical methods to obtain bioactive peptides from protein-rich sources is through protein hydrolysis, typically by using microbial fermentation or the direct addition of specific proteases [19]. Most of the bioactive peptides with a presumed role in chronic disease prevention have been found in dairy products and legumes [19]. Despite fewer studies on the bioactive potential of WF protein fractions and their hydrolysates, a comprehensive analysis of the amino acid sequences of the proteins of four cereal grains concluded that wheat and barley have the greatest abundance and diversity of potential bioactivity, with high occurrence frequencies of the peptide sequences associated with ACE inhibition and antioxidant activity, among other activities [20]. In the present study, we provide evidence, for the first time, that protein fractionation and proteolysis by digestive endopeptidases are valuable tools for functionalizing WF proteins. The research objectives were (i) to assess the molecular, functional and bioactive properties of the protein fractions from white WF; and (ii) to determine the modifications to bioactive properties, mainly antioxidant capacity, anti-ACE and anti-α-amylase activities, induced by the action of pepsin and combined trypsin–chymotrypsin in vitro.

## 2. Results and Discussion

### 2.1. Molecular Characteristics of Wheat Flour Proteins and Protein Fractions

**Figure 1:**
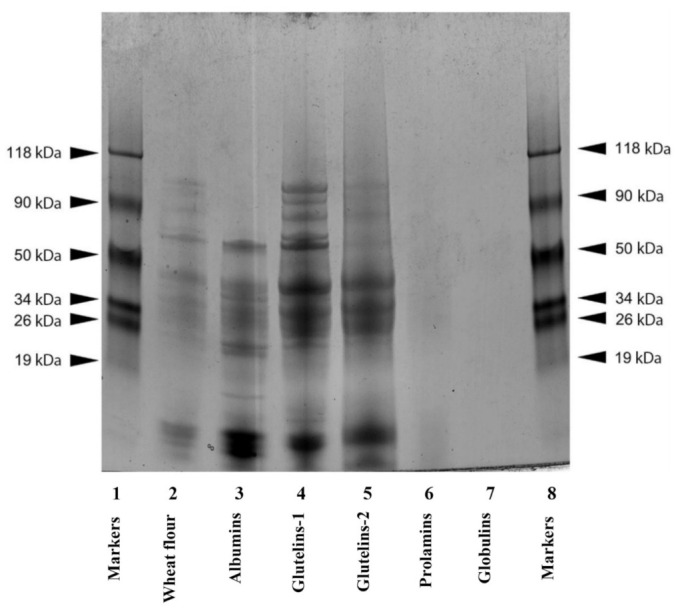
SDS-PAGE electropherogram of wheat flour proteins (lane 2), wheat flour protein fractions (albumins, lane 3; glutelins-1, lane 4; glutelins-2, lane 5; prolamins, lane 6; and globulins, lane 7) and molecular weight markers (lanes 1 and 8).

Representative SDS-PAGE patterns of the WF proteins and Osborne protein fractions are illustrated in Figure 1. The WF proteins consisted of 13 minor bands with relatively low densitometric intensity (<50%) and molecular weights (MWs) of 100.8, 95.9, 84.4, 67.5, 61.8, 43.0, 39.5, 35.3, 28.7, 23.2, 9.7, 8.9 and 8 kDa, respectively. In contrast, the protein components of the albumin fraction were characterized by a lower MW (<60 kD), and were distributed into seven major bands (56.4, 39.4, 30.8, 21, 9.6, 8.6 and 8.3 kDa) and seven minor bands (36.4, 26.8, 25, 20, 16.7, 13.6 and 10.9 kDa). Similarly, the glutelins-1 consisted of five major bands (56.5, 39.6, 29.4, 25.2 and 9.4 kDa) and eight minor bands (87.3, 81.1, 77.7, 61.6, 23.5, 21.6, 18.3 and 11.3 kDa), while the glutelins-2 consisted of four major bands (39.8, 29.3, 25.8 and 11.2 kDa) and eight minor bands (98.9, 93.9, 80.3, 58.2, 23.4, 20.5 and 8.2 kDa). One minor band (26.4 kDa) was detected in the prolamins, while no bands were found for the globulins, possibly due to the relatively low protein content of this fraction (0.46%) as compared to the other fractions (albumins, 28.5%; prolamins, 32.8%; glutelins-2, 43.5%; glutelins-1, 71.0%). The total protein content of the flour (10.4%) was within the range found for different wheat cultivars (9.3–12.6%) [21].

The broad range of MWs detected in this study concurs with the presence of low- (*ca.* 10–55 kDa), medium- and high-MW (*ca.* 70–140 kDa) protein types previously reported in wheat grains and flour, particularly in the glutelin fraction, which consists of low- and high-MW subunits [7,22], and in the prolamin fraction, where high-MW prolamins can be present in a small proportion [9]. In our work, low- and high-MW subunits were evidenced both in the glutelin-1 and glutelin-2 fractions. The relatively low MW of the band detected for the prolamins (26.4 kDa) is consistent with the monomeric nature of most of the gliadins found in wheat prolamins (e.g., α-gliadins) [9,22] and with the MW range of α-gliadins (*ca.* 30–45 kDa) [9,23]. For the albumins, our findings concur with the MW range of 5–75 kDa reported previously [21]. According to Schalk et al. [7], albumins/globulins are the least abundant in WF (1.22 g/100 g flour) as compared to glutelins and prolamins (2.98 and 5.94 g/100 g, respectively), which may hinder the recovery of globulins as noted previously [24], and could explain the relatively low protein content and yield (0.46% and 2.04%) of globulins in the present work. Similar findings were presented by Hailegiorgis et al. [25], who reported that globulins accounted for less than 10% of the total proteins in durum wheat varieties, with MWs from 10 to 70 kDa. In rice, the protein yield of globulins (3.85%) was amongst the lowest values as compared to those of glutelins and albumins [26].

### 2.2. Amino Acid Profile of Wheat Flour Proteins and Protein Fractions

The amino acid (AA) composition of the WF proteins and protein fractions (Table 1) revealed important characteristics in terms of the proportions and distribution of essential vs. non-essential and polar vs. nonpolar AAs. Cysteine, glutamic acid and leucine were most abundant in WF and in the albumin, glutelin-1 and glutelin-2 fractions, with mean concentrations ranging from 22.71 to 31.03 g/100 g, 12.81 to 14.47 g/100 g and 9.37 to 11.3 g/100 g, respectively, in these proteins. The relatively high content of cysteine is noteworthy, as these residues can form inter- and intrachain disulfide bonds [9,27] that have a high relevance for designing functional and bioactive proteins and peptides [28]. Another biologically important attribute of cysteine is that its side chain exhibits both polar and hydrophobic characteristics [29]. Likewise, leucine, an essential branched-chain and hydrophobic AA, plays important roles in human physiology that impact the risk of several chronic diseases [30].

In contrast, the prolamins exhibited a high proportion of polar AAs, including glutamic acid, aspartic acid and serine (20.90, 7.86 and 6.37 g/100 g, respectively). Both glutamic acid and proline (not reported in this study) are generally the predominant AAs in wheat prolamins [9,31]. Overall, our findings corroborate earlier reports about prolamins [31] and WF [32] AA composition. In terms of limiting essential AAs, the small amounts of threonine, phenylalanine, isoleucine and methionine in the prolamins, and of methionine, lysine and isoleucine in the other fractions, are in general agreement with previous reports [31,32,33]. Tryptophan was not reported in most studies nor in our work because it is easily destroyed during AA analysis [34]. The AA content of the globulin fraction could not be determined due to the low protein content of this fraction.

### 2.3. Functional Properties of Wheat Flour and Its Protein Fractions

This study is the first to comparatively assess the functional properties of the individual protein fractions from WF in terms of water holding capacity (WHC), foam stability and emulsion stability. As shown in Table 2, the prolamins exhibited the second-highest WHC (53.6%) after WF (84.4%), followed by glutelins-1 (37.6%) and glutelins-2 (22.0%). The relatively high WHC of the prolamins is in keeping with the AA composition of this fraction, specifically its high proportion of polar hydrophilic AAs, and with other important functional characteristics of wheat gliadins, namely their solubility in water at low concentrations, their ability to form gel-like hydrated nanostructures at greater concentrations [22], and their plasticizing effect, which improves the viscosity and extensibility of gluten proteins in dough [35]. In contrast, the albumin and globulin fractions exhibited a significantly lower WHC (6.0%) (*p* < 0.05), possibly due to their distinct AA composition and relatively low protein content. While proteins play an important role in WHC by interacting with water and other solutes, other molecules, such as carbohydrates and phenolic compounds which may be present in the protein extracts, could also contribute to their water-binding capacity. For WF, our finding is consistent with the high WHC (*ca.* 90%) reported by Mesias and Morales [36]. In other studies, wheat protein isolates (WPI) were found to exhibit high water retention [37] and a greater WHC compared to sodium caseinate, nonfat dry milk and dried egg whites [38].

Superior foam stability was achieved by the albumins (76.4%), followed by the glutelins-2 (41.4%) and prolamins (31.1%). Much lower values were obtained with WF, glutelins-1 and globulins (18.3, 16.2 and 5.8%, respectively). Echoing our findings, Hassan et al. [37] evidenced greater foam stability with wheat albumins compared to WPI at pH values above 6.0, while Yang et al. [39] showed that the foaming properties of albumins from yellow beans, mung beans and Bambara groundnuts were superior to those of globulins. Albumins’ superiority as a foaming ingredient has been attributed to its high water solubility, which is important for foam formation, together with its ability to form strong interfacial layers around air bubbles [37,39]. Using bovine serum albumin as a model protein, Han et al. [40] showed that conformational changes at specific locations in the primary structure upon adsorption at the air/water interface could induce structural changes in the surrounding peptides, which could lower the α-helix content of the adsorbed protein. In contrast, it has been suggested that the extraction of plant globulins results in aggregated structures that prevent the formation of strong interfacial layers in foams [39]. According to our findings, the albumins, glutelins-2 and prolamins from WF all possess enhanced foam stabilizing properties as compared to unfractionated proteins, and may thus prove useful as foaming ingredients.

The glutelins-1, albumins and WF each conferred a relatively high emulsion stability (50.0, 43.9 and 41.9%, respectively). Significantly greater emulsion stability (*p* < 0.05) was achieved with glutelins-1 than with glutelins-2, prolamins or globulins. In partial agreement with our findings, Hassan et al. [37] showed greater emulsion stability with wheat albumins than with WPI. The structural features that enable the extracted albumins and glutelins-1 to stabilize the oil–water interface remain to be elucidated. It can be surmised that partial protein unfolding at the interface and the exposure of hydrophobic patches both play a crucial role. Based on our findings, the WF protein fractions with the greatest potential as functional ingredients are the prolamins and glutelins-1 (WHC), albumins and glutelins-2 (foaming capacity) and glutelins-1 and albumins (emulsifying capacity). Their foaming and emulsifying properties could be studied further to establish the underlying molecular mechanisms and optimize their functionality in different food matrices.

### 2.4. Bioactive Properties of Wheat Flour and Its Protein Fractions as Modified by the Action of Trypsin and Chymotrypsin

In this first report of the effects of protein fractionation and proteolysis by trypsin–chymotrypsin (TC) and pepsin (P) on the biological activities of WF proteins, each protein fraction and its hydrolysates were evaluated for antioxidant activity and inhibitory activity against ACE and α-amylase, before and after 3 h of hydrolysis with TC (TC-H) or P (P-H) at a pH of 8.0 or 2.0, respectively. In addition to the antioxidant activity assessed using diphenyl-1-picrylhydrazyl (DPPH) radical scavenging activity, the total content of the antioxidant compounds was determined using the Folin–Ciocalteu method reagent, which reacts with diverse classes of antioxidant compounds, including aromatic and thiol-containing aromatic AAs, peptides and phenolic compounds [41]. As shown in Table 3, the highest content of antioxidant compounds was found in the glutelins-1 before TC-H (8.80 mg GAE/g), followed by the albumins, glutelins-2 and WF (5.16, 4.59 and 4.46 mg GAE/g, respectively). After TC-H, the total antioxidant content of the WF, glutelin-1 and glutelin-2 hydrolysates reached comparable levels around 11 mg GAE/g as the total antioxidant content of the WF and glutelins-2 increased significantly, by more than 2-fold (*p* < 0.05), following TC-H (Table 3, middle and lower panels). Antioxidant activity was the highest in the WF and prolamins (17.14 and 16.54%), followed by glutelins-1 and globulins (11.14 and 10.02%) before TC-H. Similarly, prolamin hydrolysates showed the highest antioxidant action after TC-H (14.74%), followed by the glutelin-1 and globulin hydrolysates (12.23 and 11.70%). Moderate-to-high ACE inhibition (47–70%) was obtained across all the samples. The globulins exhibited the highest inhibitory activity against this enzyme both before and after TC-H (70.64 and 69.67%, respectively), together with the prolamin hydrolysates after TC-H (70.08%). A relatively high ACE inhibition was also noted with glutelins-1 and WF (*ca.* 66%) before TC-H, as well as in the glutelins-1 hydrolysates (67.80%). The fractions with superior α-amylase inhibitory activity were WF, globulins and prolamins both before and after TC-H (97.22 vs. 93.52%, 94.45 vs. 93.52% and 93.52 vs. 97.22%, respectively), followed by glutelins-1 (73.16 vs. 75.93%). As summarized in Table 4, the anti-α-amylase and anti-ACE activities of the TC hydrolysates were mutually correlated (*r* = 0.891, *p* < 0.05) and positively associated with antioxidant activity (*r* = 0.583 and 0.812, respectively, *p* < 0.05).

These novel findings advance the understanding of the multiple biological activities associated with WF proteins. In particular, the globulins, prolamins, glutelins-1, WF and their TC hydrolysates showed a high capacity to inhibit both ACE and α-amylase, which may be attributed to the presence of inhibitory peptides and other phytochemicals, notably phenolic compounds, as suggested by studies of soybeans and other plant-based proteins [42,43,44]. These compounds could either bind to the enzyme active sites, or chelate the zinc ion or calcium and chloride ions (cofactors) which are essential to the catalytic activity of ACE [45] and α-amylase [46], respectively. Consistent with our results, Mousavi et al. [47] found relatively high inhibitory activity against α-amylase (53.3%) in the lowest MW peptide fraction (<1 kDa) obtained after the hydrolysis of soft wheat prolamins with actinidin, especially in one subfraction (71.2%), possibly due to its high levels of proline and AAs with hydroxyl groups. The low anti-α-amylase activity of the albumin fraction in the present work suggests that the α-amylase inhibitors that naturally occur in the water-soluble fraction of wheat proteins [48] may be inactive at a pH of 8.0. According to Zhang et al. [11], ACE inhibition by wheat gluten after alcalase treatment depends on the wheat gluten structure before enzymolysis, including the α-helix, random coil and free sulfhydryl contents, which could explain why the different protein fractions exhibited different degrees of ACE inhibition in our work. ACE inhibition is generally attributed to short-chain peptides with fewer than 12 AA residues, as larger peptides cannot bind to the enzyme active sites [45]. The fact that two endopeptidases were used in our study means that the hydrolysates were enriched with polypeptides and peptides rather than with free AAs. The possible role of the phenolic compounds may be inferred from the findings of Alu’datt et al. [49], who showed that the phenolic extracts from barley protein fractions exerted anti-ACE, anti-α-amylase and antioxidant activities, especially those extracted from the prolamin fraction (*ca.* 84%, 70% and 61%, respectively). Echoing our findings, they found the highest ACE inhibition (61%) and antioxidant activity (77%) with barley prolamins following pepsin–trypsin hydrolysis [49]. 

In our study, the increased content of total antioxidant compounds after TC-H suggests a higher reactivity towards the Folin–Ciocalteu reagent, possibly due to the release of active peptides and free AAs, as well as conformational rearrangements leading to a greater exposure of the antioxidant sites in the (poly)peptide chains, some of which may be mediated by protein–phenolic interactions. The trend towards increased antioxidant activity after TC-H, except for WF and prolamins, is in partial agreement with the enhanced free radical scavenging activity reported by Zhao et al. [10] after the fermentation of wheat gluten by *Bacillus subtilis*, an important producer of alkaline proteases. Our findings of significant positive associations between the antioxidant, anti-α-amylase and anti-ACE activities of TC hydrolysates have high practical relevance. Indeed, they indicate that protein/peptide fractions with multiple health-promoting bioactivities can be obtained through fractionation and TC-catalyzed hydrolysis. Future studies could help elucidate the molecular mechanisms that govern the antioxidant potency of WF protein/peptide-based fractions and their inhibition of ACE and α-amylase.

### 2.5. Bioactive Properties of Wheat Flour and Its Protein Fractions as Modified by the Action of Pepsin

The biological properties of WF protein fractions and their peptic hydrolysates are presented in Table 5. The albumins showed the highest total antioxidant content before P-H (61.92 mg GAE/g), followed by the glutelins-1 (48.84 mg/g). P-H significantly enhanced the antioxidant content of glutelins-1, which reached the highest value after P-H (62.36 mg/g), while the amount of antioxidant compounds in the albumin, WF and glutelin-2 hydrolysates was reduced significantly after P-H (Table 5, middle and lower panels). A superior antioxidant capacity was obtained with the prolamins before and after P-H (94.18 vs. 95.08%), followed by the glutelins-1 (68.44 vs. 66.07%) and globulins (57.71 vs. 59.84%). Moderate-to-high inhibition of ACE (39–89%) was evidenced in all the samples. The strongest inhibition was with WF before P-H (85.14%), followed by glutelins-2 (77.20%), then albumins, glutelins-1 and prolamins (66.67–70.10%). ACE inhibition by the globulins increased significantly to 89.02% after P-H, exceeding the anti-ACE activity of the hydrolyzed WF and glutelins-2 (77.87 and 77.37%), as well as the albumins and prolamins (72.47 and 71.07%). Prolamins possessed superior anti-α-amylase activity before P-H (71.49%), together with the albumin hydrolysates (74.49%). WF, globulins and albumins before P-H, and the globulin hydrolysates, also showed appreciable anti-amylase action greater than 45%. No significant correlations were found between the biological properties of the P hydrolysates, except for ACE inhibition, which was inversely associated with the total antioxidant content (*r* = –0.878, *p* < 0.05, Table 6).

These findings lend further support to the effectiveness of protein fractionation and proteolytic processes as strategies to optimize the biological activities of WF proteins in a way that prompts the development of nutraceutical and therapeutic applications targeting diabetes and hypertension, among other conditions. In contrast to the other protein fractions, the prolamins possessed relatively high antioxidant and anti-ACE activities both before and after P-H, while pepsinolysis was required for the globulins and albumins to potently inhibit ACE and α-amylase, respectively. In line with our findings, a study on barley protein fractions showed that the prolamin fraction displayed the highest antioxidant and anti-ACE activities (61% and 77%, respectively) after combined pepsin and trypsin hydrolysis [49]. The diverse bioactivity profiles evidenced for each fraction could be explained not only by the primary and higher structures of their protein and peptide components, but also by the presence of other naturally occurring phytochemicals, such as phenolic compounds which are often associated with plant proteins, including wheat proteins [50]. In Gammoh et al.’s study [50], the amount of total phenolics extracted from wheat prolamins and the antioxidant activity of these phenolics were strongly positively correlated, while the total phenolic content and antioxidant activity of the phenolic compounds from barley proteins were found to be weakly correlated [49]. The absence of significant correlations between the total antioxidant content and antioxidant activity in the current study could be related to the fact that the extracts that were characterized are protein extracts, rather than phenolic extracts. The protein extracts probably consisted of a complex mixture of protein/peptides with smaller amounts of phenolic compounds.

An important contribution of this study is the identification of WF protein fractions and hydrolysates with high potential as sources of antioxidants and natural ACE and α-amylase inhibitors. The synopsis provided in Table 7 highlights the potentialities of each protein/peptide-based fraction in terms of its antioxidant capacity and inhibitory activities against ACE and α-amylase. It demonstrates the broad spectrum of bioactivities that can be derived from WF proteins through fractionation and hydrolysis by trypsin–chymotrypsin or pepsin. Regardless of the proteolytic treatment, the prolamins, glutelins-1 and globulins were consistently associated with relatively high antioxidant activity. Moreover, these fractions displayed multiple bioactivities, the extent of which was influenced by proteolytic treatment.

### 2.6. Comparison of the Bioactive Properties Fractions Resulting from Each Treatment

A comparison of the biological activities resulting from each treatment is shown in Table 8 as ratios of the values obtained before or after P-H versus TC-H. For the total antioxidant content and antioxidant activity pre-hydrolysis, the ratios generally exceeded 1, indicating that the values obtained at pH 2.0 before P-H were greater than at a pH 8.0 before TC-H. This difference was statistically significant (*p* < 0.05) in terms of the total antioxidant content of WF, albumins, glutelins-1, glutelins-2 and globulins (P/TC ratios ranging from 5.31 to 12.0), as well as antioxidant activity of glutelins-1, prolamins and globulins. The exception was glutelins-2 antioxidant activity, which was significantly lower before P-H than before TC-H (ratio of 0.12). A similar trend was evidenced after hydrolysis, except that a statistical significance was found only with the glutelins-1, prolamins and globulins. For most of the fractions, ACE inhibition also tended to be greater pre- and post-P-H as compared to TC-H. However, none of these differences were significant. In contrast, the values of α-amylase inhibition tended to be superior with the TC treatment (P/TC ratios < 1). This was significant for glutelins-1 and -2 pre-hydrolysis (ratios of 0.23 and 0.09), and post-hydrolysis for prolamins (0.22) (*p* < 0.05). A notable exception was the albumin fraction, which showed a significantly higher anti-α-amylase activity pre- and post-P-H versus TC-H (ratios > 7), suggesting that acidic conditions play a key role in the formation and stabilization of bioactive sites in these proteins and their derived peptides. In model albumin proteins, acid expansion of the protein below pH 3.5 results in newly exposed surface patches [51] and greater accessibility of the C-terminal moiety where cleavage by pepsin preferentially occurs [52]. These proteins undergo a distinct conformational transition at pH values between 7.0 and 9.0 [51,53]. The presence of α-amylase inhibitors in the albumin fraction could provide an additional explanation. These inhibitors are considered to be most abundant in cereal albumins [48]. An endogenous α-amylase inhibitor extracted from wheat seeds was found to have an optimum activity at a pH of 5.1, according to Warchalewski [54].

Our findings provide new practical and fundamental insights into the conditions that enhance the biological activities of WF protein fractions. We posit that the treatment pH (2.0 vs. 8.0) and the distinct modes of action of pepsin versus trypsin and chymotrypsin at their optimal pH are important drivers of the molecular rearrangements that govern the bioactivity of these proteins/peptides and their associated compounds. Fu et al. [55] showed that pepsin is more efficient than trypsin and chymotrypsin at cleaving native or tightly folded proteins, possibly due to the denaturing conditions, and thus has greater accessibility to internal cleavage sites at low pH values. Pre-digestion by pepsin at low pH greatly increases the proteolytic efficiency of trypsin and chymotrypsin [55,56]. The use of peptic hydrolysates could be particularly attractive for foods and other applications with an acidic pH.

## 3. Materials and Methods

### 3.1. Plant Material and Chemicals

Commercial white wheat flour (WF) was obtained from a local market in Amman, Jordan. Composite samples were prepared from 25 bags of 1.5 kg each. Two subsamples of 50 g each were collected from each bag, then pooled and carefully mixed before transferring into sealed plastic bags that were kept at 4 °C until further use. This sampling method was used to prepare representative samples [57]. WF proximate composition (78.5% total carbohydrates, 10.4% crude protein, 1.5% fat, 9% water and 0.6% ash) was determined in accordance with the standard procedures of the Association of Official Analytical Chemists [58] (AOAC 992.23 for crude protein, AOAC 948.15 for fat, AOAC 952.08 for moisture and AOAC 930.30 for ash). The total carbohydrate content was calculated by subtracting the sum of the other contents from 100. All the reagents used were of analytical grade and supplied by Sigma-Aldrich (Saint-Louis, MO, USA). HPLC-grade solvents and amino acid standards were used for ion-exchange chromatography. ACE from rabbit lungs (CAS No. 9015-82-1, product No. 1134007), α-amylase from porcine pancreas (9000-90-2, A3176), chymotrypsin and trypsin from bovine pancreas (9004-07-3, C4129; 9002-07-7, T1426) and porcine pepsin (9001-75-6, P7012) were also purchased from Sigma-Aldrich.

### 3.2. Fractionation of Wheat Flour Proteins

WF proteins were fractionated using the sequential extraction scheme of Osborne reported by Kwon et al. [59], and adapted by Gammoh et al. [50]. Full-fat WF (10 g) was extracted first by adding distilled water (100 mL) and stirring at room temperature (25 °C) for 1 h. The insoluble residue was removed using centrifugation at 10,000× *g* for 15 min at 4 °C, while the supernatant was filtered through a cheesecloth, lyophilized (LFD-5508 freeze drier, Daihan Labtech Co., Kyonggi-do, Republic of Korea) and stored at −18 °C until further analysis. This procedure was repeated with four different extraction solvents, namely NaOH (0.1 M), glacial acetic acid (50%), ethanol (80%) and NaCl (10%), using a 1:10 (*w*/*v*) residue-to-solvent ratio to dissolve the previously insoluble residue. The protein fractions extracted with distilled water, NaOH, acetic acid, ethanol and NaCl were respectively labeled as the albumin, glutelin-1, glutelin-2, prolamin and globulin fractions. The extraction procedure was replicated twice and the samples were pooled. The average protein content and yield of each protein fraction, determined according to Gammoh et al. [60] and expressed in g/100 g on a dry weight basis, were 28.5% (protein yield 6.97%), 71.0% (21%), 43.5% (3.57%), 32.8% (0.43%) and 0.46% (2.04%), respectively.

### 3.3. Protein Molecular Characterization with SDS-PAGE

The molecular characterization of the WF proteins and protein fractions was performed using sodium dodecyl sulfate-polyacrylamide gel electrophoresis (SDS-PAGE), using Laemmli’s method [61] as described by Alu’datt et al. [62]. The WF or lyophilized protein fractions (3 mg) were dissolved in 1 mL of a solubilizing buffer, containing 0.5 mL of β-mercaptoethanol, 1 mL of bromophenol blue (1%), 0.6 mL of Tris-HCl (1 M, pH 6.8), 2 mL of 10% SDS (10%), 5 mL of glycerol (50%) and 0.9 mL of distilled water, followed by denaturation in a boiling water bath for 3 min with stirring. Electrophoresis was performed with 4–20% Mini-Protean TGX Precast Gels in a Mini-Protean Tetra Cell (Bio-Rad Laboratories, Hercules, CA, USA), with a migration buffer containing SDS (0.1%), glycine (0.192 M) and Tris-HCl (0.025 M, pH 8.3), and a migration voltage increasing from 60 to 120 V. The gels were stained with a solution of modified Coomassie Brilliant Blue G 250, destained and dried before scanning and analysis (Bio-Rad GS-800 densitometer and Quantity One 1-D Analysis Software, respectively). The molecular weight (MW) of the proteins was determined from a standard curve of the migration distance of MW markers (19–118 kDa) vs. the log of MW. Their corresponding bands were categorized as major or minor bands based on the densitometric intensity (greater or less than 50%, respectively).

### 3.4. Amino Acid Determination

The amino acid content of the WF proteins and protein fractions was determined using ion-exchange HPLC after the complete acid hydrolysis of the proteins, as previously described [63,64]. The proteins were hydrolyzed by mixing 1 g of the sample with 10 mL of 6 M HCl and heating at 100 °C for 24 h under vacuum conditions in a distillation unit. The supernatant was filtered (0.45 mm membrane filter), lyophilized and then kept at −18 °C until assayed. The HPLC was conducted with a Sykam S 433 automatic amino acid analyzer (Sykam GmbH, Eresing, Germany), equipped with a cation-exchange column (LCA K07/Li, 4.6 × 150 mm, Sykam GmbH) and a dual-channel photometer. The hydrolyzed samples and amino acid standards were diluted in 1 mL of sodium citrate buffer (0.12 M, pH 2.2) before injection (100 μL) into the system. The eluent (sodium acetate 90% and acetonitrile 10%) and ninhydrin solution were delivered at 0.7 mL/min into the reaction coil, where the mixture was heated at 130 °C for 2 min. The final reaction products were monitored at 440 nm and 570 nm, followed by peak integration. The amino acid content was expressed as g/100 g protein on a dry weight basis.

### 3.5. Functional Properties of Wheat Flour Proteins and Protein Fractions

The water holding capacity (WHC), foaming capacity and emulsifying capacity were determined according to established methods [65,66,67], with a few modifications [62] as summarized below. WHC was determined using centrifugation after inducing gel formation. The WF or lyophilized protein fraction (16 g) was mixed with 100 mL of phosphate buffer (pH 7.0) and heated at 95 °C for 30 min to induce gelation. The gels were cooled and kept at 4 °C for 24 h before centrifugation at 10,000× *g* for 15 min at 4 °C in a benchtop centrifuge (Thermo Fisher Scientific, Cambridge, MA, USA). The volume of supernatant (V_s_) collected after centrifugation was measured and the WHC (%) was expressed as the percentage of liquid retained in the gel relative to the initial volume of liquid (V_T_), according to Equation (1):

WHC (%) = ([V_T_ − V_s_]/V_T_) × 100 (1)

For foam stability, 2 g of the sample and 40 mL of distilled water were mixed for 5 min at 1600 rpm with an immersion hand blender (Braun GmbH, Kronberg, Germany) in a 100 mL graduated cylinder at room temperature (25 °C). The total volume of foam initially (V_T_) and after 60 min (V_1_) at room temperature were measured, and the foam stability (%) was calculated using Equation (2):

Foam stability (%) = (V_1_/V_T_) × 100 (2)

For emulsion stability, 2 g of the sample, 20 mL of olive oil and 20 mL of distilled water were blended for 2 min at 1600 rpm at ambient temperature using a hand blender, and the total height (H_T_) of the resulting emulsion was recorded. The height of the emulsified layer was measured again (H_1_) after heating the emulsion at 80 °C for 30 min in a water bath in a 50 mL graduated centrifuge tube (Falcon, Thermo Fisher Scientific), cooling for 15 min under tap water and centrifuging in a benchtop centrifuge (2000× *g*, 4 °C, 15 min). The emulsion stability (%) was calculated as follows (Equation (3)):

Emulsion stability (%) = (H_1_/H_T_) × 100 (3)

### 3.6. Enzymatic Hydrolysis of Wheat Flour Proteins and Protein Fractions by Digestive Proteases

Pepsinolysis was performed according to the procedure described by Megias et al. [68], with the following modifications. One gram of sample was mixed with pepsin (enzyme-to-protein ratio of 1:20 *w*/*w*) in 100 mL acidic distilled water (0.1 M HCl, pH 2.0). An aliquot of this mixture (3 mL in three test tubes of 1 mL each) was immediately heated at 95 °C for 5 min in a shaking water bath to inactivate the enzyme, representing time 0 (pre-hydrolysis) at a pH of 2.0. After centrifugation (10,000× *g*, 10 min, 4 °C), the supernatant was filtered through a cheesecloth, cooled and kept at −18 °C until subsequent analysis for biological activities. The remaining portion of the sample and enzyme mixture was incubated for 3 h at 37 °C in a shaking water bath, followed by heat inactivation of the enzyme, centrifugation and freezing as previously described. For hydrolysis with trypsin and chymotrypsin (1:1 *w*/*w* mixture), the same procedure was followed with an enzyme-to-protein ratio of 1:20 *w*/*w*, except that alkaline distilled water (0.1 M NaOH, pH 8.0) was used in the first step.

### 3.7. Biological Properties of Hydrolyzed and Unhydrolyzed Wheat Flour Proteins and Protein Fractions

#### 3.7.1. Total Antioxidant Compounds

The total concentration of antioxidant compounds, including aromatic and thiol-containing aromatic amino acids, peptides and phenolic compounds [41], was measured using the Folin–Ciocalteu spectrophotometric method reported by Gammoh et al. [69], with the following modifications. The hydrolyzed or unhydrolyzed protein samples (0.1 mL) were mixed with distilled water (8.4 mL), followed by the addition of Folin–Ciocalteu reagent (0.5 mL), vortexing for 4 min and addition of a sodium carbonate solution (1 mL, 5% *w*/*v*). The mixture was incubated at ambient temperature for 60 min in the dark before reading the absorbance at 725 nm in duplicate (UV 1800 spectrophotometer, Biotech Engineering Management Co., Nicosia, Cyprus). The content of the antioxidant compounds was derived from a standard curve prepared with gallic acid and expressed in mg of gallic acid equivalents (GAE) per g of dry weight.

#### 3.7.2. Antioxidant Activity

The 2,2-diphenyl-1-picrylhydrazyl (DPPH) assay for antioxidant activity was adapted from Brand-Williams et al. [70]. The hydrolyzed or unhydrolyzed protein samples (0.1 mL) were mixed with 3.9 mL of 6 × 10^–5^ M DPPH solution, prepared by dissolving 2.4 mg of DPPH dissolved in 100 mL of methanol. The mixtures were incubated for 30 min at room temperature in the dark and centrifuged (1400× *g*, 15 min, 4 °C) before reading the absorbance of the supernatant at 515 nm. The absorbance was measured in duplicate after 30 min for each sample (A_S30_) and initially (A_B0_) for a blank consisting of DPPH in methanol. Antioxidant activity (%) was calculated as follows (Equation (4)):

Antioxidant activity (%) = ([A_B0_ − A_S30_]/A_B0_) × 100 (4)

#### 3.7.3. ACE Inhibitory Activity

ACE inhibition was measured according to the method of Cushman and Cheung [71], with modifications. The enzyme solution was prepared by mixing ACE (0.33 unit/mL) with 1 mL of distilled water. For the substrate, a solution of hippuric-L-histidyl-L-leucine (HHL, 0.3% (*w*/*v*)) was prepared in HEPES (4-(2-hydroxyethyl)-1-piperazineethanesulfonic acid) HCl buffer (50 mM, pH 8.3) containing NaCl (300 mM) in distilled water. The test samples (100 μL) or blanks (100 μL of distilled water) were mixed with 200 μL of HHL solution and 50 μL of ACE solution and incubated for 15 min at 37 °C in a shaking water bath. After 15 min, the enzymatic reaction was ended by adding 250 μL of HCl 1.0 M. The hippuric acid released by the enzymatic hydrolysis was collected from the solution by mixing with 2 mL of ethyl acetate, followed by centrifugation (1400× *g*, 3 min, 4 °C). One mL of the ethyl acetate layer was pipetted and evaporated by boiling in a shaking water bath at 100 °C for 15 min. Finally, 3 mL of distilled water was added, and the absorbance of the samples (A_S_) and blanks (A_B_, no inhibition of ACE) was monitored at 228 nm in duplicate to detect the hippuric acid released. ACE inhibition (%) was expressed as the percentage decrease in enzyme activity relative to the blank, using Equation (5):

ACE inhibitory activity (%) = ([A_B_ − A_S_]/A_B_) × 100 (5)

##### 3.7.4. α-Amylase Inhibitory Activity

The inhibition of pancreatic α-amylase was determined according to McCue et al. [72], with a few adjustments. The enzyme solution (0.03% *w*/*v*) was prepared by dissolving α-amylase (30 mg) in 100 mL of distilled water. The substrate (0.5% *w*/*v* starch solution) was prepared by mixing potato starch (0.125 g) in a phosphate buffer (25 mL, pH 7.0) with continuous stirring at 65 °C for 20 min. The test samples (100 μL) or blanks (100 μL of distilled water) were mixed with 500 μL of starch solution and 500 μL of α-amylase solution, followed by incubation at 25 °C for 10 min in a shaking water bath. Next, 1 mL of the colorimetric reagent (mixture of 3,5-dinitrosalicylic acid (DNS, 10.6 g), NaOH (19.8 g), phenol (7.6 mL), sodium metabisulfite (8.3 g) and sodium potassium tartrate (3.06 g) in 1416 mL of distilled water) was added, followed by heating (95 °C for 5 min in a shaking water bath) and then cooling to ambient temperature in an ice bath. The mixture was diluted to 10 mL by adding distilled water before reading the absorbance of the samples (A_S_) and blanks (A_B_, no inhibition of α-amylase) at 540 nm in duplicate to detect the quantity of maltose formed. The inhibition of α-amylase (%) was expressed as the percentage decrease in enzyme activity relative to the blank (Equation (6)):

α-amylase inhibitory activity (%) = ([A_B_ − A_S_]/A_B_) × 100 (6)

### 3.8. Statistical Analyses

All the experiments were carried out in duplicate. Statistical analyses were conducted with the general linear model procedure and SAS statistical package (v. 9.4, 2018, SAS Institute Inc., Cary, NC, USA). Data were checked for normality and equality of variance. One-way analysis of variance (ANOVA) followed by least significant difference (LSD) multiple comparison tests were performed to separate treatment means. The associations between the bioactive characteristics (antioxidant activity, total antioxidant compounds, ACE and α-amylase inhibition) were assessed using Pearson’s correlation coefficients (*r*). Statistical significance was inferred at *p* < 0.05.

## 4. Conclusions

This study demonstrates the value of protein fractionation and proteolytic processes involving gastrointestinal proteases for functionalizing wheat flour proteins and generating protein/peptide-enriched extracts with enhanced functional and biological properties. The molecular characterization of the most abundant protein fractions showed that the albumins and glutelins consisted of a mixture of low- and high-MW polypeptides rich in cysteine, glutamic acid and leucine, while low-MW protein components rich in polar amino acids prevailed in the prolamins. These fractions displayed the greatest potential as functional ingredients with high-to-moderate WHC (prolamins and glutelins-1), foaming capacity (albumins and glutelins-2) and emulsifying capacity (glutelins-1 and albumins). Bioactivity profiling, before and after pepsin or trypsin–chymotrypsin hydrolysis, revealed that the protein fractions, including the globulins, each possessed a unique bioactivity potential, which often outperformed that of the unfractionated proteins in terms of antioxidant capacity and/or inhibition of α-amylase and ACE, either before and/or after proteolysis. The prolamins, globulins, glutelins and their hydrolysates were amongst the most promising fractions in that regard. These novel findings show that wheat protein fractions and their peptide-enriched P and TC hydrolysates are promising sources of multifunctional bioactive ingredients that could advance the development of functional foods, nutraceuticals and natural drugs targeting diabetes and hypertension, among other conditions. Further research is recommended to identify the active components of these fractions and assess their health-promoting effects in vivo.

## Figures and Tables

**Table 1:** Amino acid composition (g/100 g protein dry weight basis) of wheat flour and its protein fractions.

Amino Acids	Wheat Flour	Albumins	Glutelins-1	Glutelins-2	Prolamins
Cysteine	23.54 ± 0.22 ^aB^	31.03 ± 1.01 ^aA^	22.98 ± 0.02 ^aB^	22.71 ± 0.07 ^aB^	0.84 ± 0.73 ^eC^
*Glutamic acid*	12.81 ± 0.09 ^bC^	13.17 ± 0.24 ^bBC^	13.55 ± 0.09 ^bBC^	14.47 ± 0.04 ^bB^	20.90 ± 1.3 ^aA^
Leucine *	11.30 ± 0.06 ^cA^	9.37 ± 0.32 ^cC^	11.10 ± 0.02 ^cA^	10.45 ± 0.03 ^cB^	2.24 ± 0.27 ^deD^
*Arginine*	6.75 ± 0.54 ^eB^	5.92 ± 0.31 ^dC^	5.90 ± 0.04 ^fC^	7.40 ± 0.05 ^dA^	0.33 ± 0.13 ^eD^
*Threonine* *	7.39 ± 0.08 ^dA^	4.68 ± 2.97 ^defA^	5.47 ± 0.03 ^gA^	6.92 ± 0.02 ^eA^	0.19 ± 0.03 ^eB^
Phenylalanine *	7.00 ± 0.17 ^eB^	5.41 ± 0.18 ^dB^	7.47 ± 0.01 ^dA^	6.69 ± 0.19 ^fAB^	1.36 ± 0.80 ^deC^
Alanine	6.28 ± 0.08 ^fA^	5.68 ± 0.19 ^dA^	6.88 ± 0.03 ^eA^	6.47 ± 0.03 ^gA^	1.45 ± 0.76 ^deB^
*Histidine* *	3.52 ± 0.02 ^hB^	4.79 ± 0.16 ^deA^	3.70 ± 0.01 ^iB^	4.77 ± 0.08 ^hA^	4.87 ± 0.18 ^deA^
*Tyrosine*	1.76 ± 0.01 ^jD^	2.04 ± 0.04 ^ghC^	2.88 ± 0.02 ^kB^	4.33 ± 0.04 ^iA^	1.11 ± 0.02 ^deE^
Valine *	6.16 ± 0.17 ^fA^	5.36 ± 0.11 ^dAB^	5.46 ± 0.01 ^gAB^	4.19 ± 0.03 ^iAB^	3.27 ± 1.88 ^deB^
Isoleucine *	5.19 ± 0.02 ^gA^	2.89 ± 0.09 ^ghC^	4.347 ± 0.03 ^hB^	3.05 ± 0.01 ^jC^	0.39 ± 0.15 ^eD^
*Lysine* *	3.57 ± 0.05 ^hB^	2.99 ± 0.14 ^fgBC^	2.95 ± 0.01 ^jBC^	2.72 ± 0.23 ^kC^	4.90 ± 0.56 ^deA^
*Serine*	2.71 ± 0.05 ^iB^	2.07 ± 0.04 ^ghC^	2.65 ± 0.03 ^lB^	2.55 ± 0.01 ^kB^	6.37 ± 0.33 ^cdA^
Methionine *	0.25 ± 0.01 ^kA^	1.17 ± 0.08 ^hiA^	2.15 ± 0.02 ^nA^	1.65 ± 0.01 ^lA^	1.63 ± 0.98 ^deA^
*Aspartic acid*	–	–	–	0.013 ± 0.04 ^mB^	7.86 ± 2.01 ^bcA^
Ammonia ^‡^	1.78 ± 0.03 ^jB^	3.47 ± 0.14 ^efgB^	2.51 ± 0.01 ^mB^	1.64 ± 0.11 ^lB^	12.38 ± 4.15 ^bA^
SE	0.121	0.586	0.023	0.063	1.753

Mean values ± standard deviation (*n* = 2 replicates), with different lowercase superscripts in a column or uppercase superscripts in a row being significantly different (*p* < 0.05). The amino acid composition of the globulins could not be determined due to the low protein content of this fraction. *Italics:* polar amino acids. *: essential amino acids; ^‡^: breakdown product of amino acids; –: not detected; SE: standard error.

**Table 2:** Functional properties of wheat flour and its protein fractions.

Wheat Flour or Protein Fractions	Water Holding Capacity (%)	Foam Stability (%)	Emulsion Stability (%)
Wheat flour	84.4 ± 0.57 ^a^	18.3 ± 2.36 ^d^	41.9 ± 3.12 ^ab^
Albumins	6.0 ± 2.81 ^e^	76.4 ± 1.96	43.9 ± 3.26 ^ab^
Glutelins-1	37.6 ± 4.26 ^c^	16.2 ± 2.75 ^d^	50.0 ± 5.40 ^a^
Glutelins-2	22.0 ± 2.83 ^d^	41.4 ± 2.02 ^b^	38.5 ± 2.12 ^b^
Prolamins	53.6 ± 2.26 ^b^	31.1 ± 3.36 ^c^	38.0 ± 2.83 ^b^
Globulins	6.0 ± 2.63 ^e^	5.8 ± 1.18 ^e^	15.6 ± 4.22 ^c^
SE	2.30	1.68	2.61

Mean values ± standard deviation (*n* = 2 replicates), with different superscripts in a column being significantly different (*p* < 0.05). SE: standard error.

**Table 3:** Total antioxidant compounds, antioxidant activity, ACE and α-amylase inhibitory activities of wheat flour and its protein fractions before and after hydrolysis with combined trypsin and chymotrypsin (TC) at a pH of 8.0, and post/pre-hydrolysis ratios of their biological properties.

Wheat Flour or Protein Fractions	Total Antioxidant Compounds (mg GAE/g)	Antioxidant Activity (%)	ACE Inhibitory Activity (%)	α-Amylase Inhibitory Activity (%)
Pre-hydrolysis (time 0 at pH 8.0)				
Wheat flour	4.46 ± 0.70 ^b^	17.14 ± 0.05 ^a^	66.29 ± 0.19 ^b^	97.22 ± 1.31 ^a^
Albumins	5.16 ± 0.03 ^b^	5.29 ± 0.79 ^d^	54.92 ± 0.04 ^d^	7.44 ± 0.05 ^d^
Glutelins-1	8.80 ± 1.19 ^a^	11.14 ± 2.20 ^b^	66.00 ± 1.42 ^b^	73.16 ± 1.31 ^b^
Glutelins-2	4.59 ± 0.83 ^b^	6.88 ± 2.14 ^cd^	63.16 ± 4.07 ^c^	62.98 ± 4.24 ^c^
Prolamins	1.32 ± 0.26 ^c^	16.54 ± 1.43 ^a^	63.83 ± 0.57 ^c^	93.52 ± 3.92 ^a^
Globulins	1.86 ± 0.25 ^c^	10.02 ± 1.11 ^bc^	70.64 ± 0.19 ^a^	94.45 ± 0.16 ^a^
SE	0.478	1.129	0.380	1.964
Post-hydrolysis with TC for 3 h				
Wheat flour	11.68 ± 0.06 ^a^	8.55 ± 0.21 ^bc^	60.04 ± 0.21 ^c^	93.52 ± 1.31 ^a^
Albumins	8.32 ± 0.58 ^b^	5.74 ± 1.56 ^c^	47.06 ± 0.09 ^e^	9.29 ± 2.62 ^c^
Glutelins-1	11.02 ± 0.03 ^a^	12.23 ± 2.23 ^ab^	67.80 ± 0.19 ^b^	75.93 ± 2.21 ^b^
Glutelins-2	11.46 ± 0.12 ^a^	9.98 ± 2.17 ^bc^	52.08 ± 0.14 ^d^	7.44 ± 2.15 ^c^
Prolamins	2.75 ± 0.29 ^c^	14.74 ± 1.86 ^a^	70.08 ± 1.33 ^a^	97.22 ± 1.31 ^a^
Globulins	2.46 ± 0.19 ^c^	11.70 ± 0.64 ^ab^	69.67 ± 1.18 ^a^	93.52 ± 1.25 ^a^
SE	0.199	1.342	0.417	1.463
Post/pre-hydrolysis ratios				
Wheat flour	**2.62**	**0.50**	**0.91**	0.96
Albumins	1.61	1.08	**0.86**	**1.24**
Glutelins-1	1.25	1.10	1.03	1.04
Glutelins-2	**2.50**	**1.45**	**0.82**	**0.12**
Prolamins	2.08	0.89	1.10	1.04
Globulins	1.32	1.17	0.99	0.99

Mean values ± standard deviation (*n* = 2 replicates), with different superscripts in a column being significantly different (*p* < 0.05). Superscripts in a column compare the protein fractions among themselves before hydrolysis (top panel) or after hydrolysis (second panel). Values of ratios in bold indicate a significant increase or decrease after trypsin–chymotrypsin hydrolysis for 3 h. ACE: angiotensin-I converting enzyme; GAE: gallic acid equivalent; SE: standard error.

**Table 4:** Correlation coefficients (*r*) between total antioxidant compounds, antioxidant activity, α-amylase and ACE inhibitory activities of trypsin–chymotrypsin hydrolysates from wheat flour and its protein fractions.

	Antioxidant Activity	α-Amylase Inhibitory Activity	ACE Inhibitory Activity
Total antioxidant compounds	–0.449	–0.438	–0.539
Antioxidant activity		0.583 *	0.812 *
α-Amylase inhibitory activity			0.891 *

* Significant correlation (*p* < 0.05). ACE: angiotensin-I converting enzyme.

**Table 5:** Total antioxidant compounds, antioxidant activity and ACE and α-amylase inhibitory activities of wheat flour and its protein fractions before and after hydrolysis with pepsin (P) at a pH of 2.0, and post/pre-hydrolysis ratios of their biological properties.

Wheat Flour or Protein Fractions	Total Antioxidant Compounds (mg GAE/g)	Antioxidant Activity (%)	ACE inhibitory Activity (%)	α-Amylase Inhibitory Activity (%)
Pre-hydrolysis (time 0 at pH 2.0)				
Wheat flour	29.51 ± 1.85 ^d^	20.98 ± 2.09 ^d^	85.14 ± 2.36 ^a^	57.98 ± 5.37 ^b^
Albumins	61.92 ± 4.76 ^a^	2.71 ± 1.50 ^e^	70.10 ± 2.87 ^c^	53.48 ± 0.28 ^b^
Glutelins-1	48.84 ± 4.52 ^b^	68.44 ± 1.04 ^b^	66.67 ± 0.69 ^c^	16.72 ± 4.31 ^c^
Glutelins-2	39.54 ± 0.82 ^c^	0.82 ± 0.23 ^e^	77.20 ± 3.20 ^b^	5.46 ± 3.24 ^d^
Prolamins	2.47 ± 1.03 ^e^	94.18 ± 1.74 ^a^	66.67 ± 1.85 ^c^	71.49 ± 4.14 ^a^
Globulins	9.88 ± 1.64 ^e^	57.71 ± 0.23 ^c^	39.36 ± 0.17 ^d^	55.73 ± 1.06 ^b^
SE	2.263	0.951	1.249	2.670
Post-hydrolysis with P for 3 h				
Wheat flour	11.92 ± 0.82 ^b^	20.66 ± 1.62 ^d^	77.87 ± 0.84 ^b^	29.47 ± 2.12 ^c^
Albumins	11.92 ± 1.23 ^b^	8.77 ± 0.35 ^e^	72.47 ± 0.87 ^c^	74.49 ± 2.10 ^a^
Glutelins-1	62.36 ± 3.91 ^a^	66.07 ± 2.09 ^b^	41.90 ± 4.86 ^d^	30.97 ± 4.24 ^c^
Glutelins-2	8.72 ± 0.01 ^bc^	7.79 ± 0.58 ^e^	77.37 ± 0.34 ^b^	22.72 ± 1.05 ^c^
Prolamins	1.60 ± 1.03 ^d^	95.08 ± 1.86 ^a^	71.07 ± 0.46 ^c^	21.22 ± 5.43 ^c^
Globulins	6.69 ± 1.64 ^c^	59.84 ± 0.23 ^c^	89.02 ± 0.90 ^a^	45.23 ± 7.55 ^b^
SE	1.330	0.955	1.204	3.614
Post/pre-hydrolysis ratios				
Wheat flour	**0.40**	0.98	**0.91**	**0.51**
Albumins	**0.19**	**3.24**	1.03	**1.39**
Glutelins-1	**1.28**	0.97	**0.63**	**1.85**
Glutelins-2	**0.22**	**9.50**	1.00	**4.16**
Prolamins	0.65	1.01	1.06	**0.30**
Globulins	0.68	1.04	**2.26**	0.81

Mean values ± standard deviation (*n* = 2 replicates), with different superscripts in a column being significantly different (*p* < 0.05). Superscripts in a column compare the protein fractions among themselves before hydrolysis (top panel) or after hydrolysis (second panel). Values of ratios in bold indicate a significant increase or decrease after pepsin hydrolysis for 3 h. ACE: angiotensin-I converting enzyme; GAE: gallic acid equivalent; SE: standard error.

**Table 6:** Correlation coefficients (*r*) between total antioxidant compounds, antioxidant activity, α-amylase and ACE inhibitory activities of pepsin hydrolysates from wheat flour and its protein fractions.

	Antioxidant Activity	α-Amylase Inhibitory Activity	ACE Inhibitory Activity
Total antioxidant compounds	0.160	–0.059	–0.878 *
Antioxidant activity		–0.388	–0.276
α-Amylase inhibitory activity			0.144

* Significant correlation (*p* < 0.05). ACE: angiotensin-I converting enzyme.

**Table 7:** Summary of wheat flour protein fractions and hydrolysates exhibiting relatively high biological activities.

Biological Activity	Pre-TC Hydrolysis (pH 8.0)	TC Hydrolysates (pH 8.0)	Pre-P Hydrolysis (pH 2.0)	P Hydrolysates (pH 2.0)
Total antioxidant compounds	Glutelins-1	WF, glutelins-1, glutelins-2	Albumins, glutelins-1	Glutelins-1, albumins, WF
Antioxidant activity	WF, prolamins, glutenins-1, globulins	Prolamins, glutelins-1, globulins	Prolamins, glutelins-1, globulins	Prolamins, glutelins-1, globulins
ACE inhibitory activity	Globulins, glutenins-1, WF	Prolamins, globulins, glutelins-1	WF, glutelins-2, albumins, glutelins-1, prolamins	Globulins, glutelins-2, WF, albumins, prolamins
α-amylase inhibitory activity	WF, globulins, prolamins, glutelins-1	Prolamins, globulins, WF, glutelins-1	Prolamins, WF, globulins, albumins	Albumins, globulins

ACE: angiotensin-I converting enzyme; P: pepsin; TC: combined trypsin and chymotrypsin; WF: wheat flour.

**Table 8:** Ratios of the biological activities of wheat flour and its protein fractions before and after pepsin (P) hydrolysis at a pH of 2.0 versus trypsin–chymotrypsin (TC) hydrolysis at a pH of 8.0.

Wheat Flour or Protein Fractions	Total Antioxidant Compounds	Antioxidant Activity	ACE Inhibitory Activity	α-Amylase Inhibitory Activity
Pre-P/Pre-TC hydrolysis ratios				
Wheat flour	**6.62**	1.22	1.28	0.60
Albumins	**12.0**	0.51	1.28	**7.19**
Glutelins-1	**5.55**	**6.14**	1.01	**0.23**
Glutelins-2	**8.61**	**0.12**	1.22	**0.09**
Prolamins	1.87	**5.69**	1.04	0.76
Globulins	**5.31**	**5.76**	0.56	0.59
Post-P/Post-TC hydrolysis ratios				
Wheat flour	1.02	2.42	1.30	0.32
Albumins	1.43	1.53	1.54	**8.02**
Glutelins-1	**5.66**	**5.40**	0.62	0.41
Glutelins-2	0.76	0.78	1.49	3.05
Prolamins	0.58	**6.45**	1.01	**0.22**
Globulins	2.72	**5.11**	1.28	0.48

Values in bold indicate significantly higher or lower values (ratio >1 or <1, respectively) before or after pepsin hydrolysis at a pH of 2.0 for 3 h, as compared to the trypsin–chymotrypsin treatment at a pH of 8.0. ACE: angiotensin-I converting enzyme.

## Data Availability

Not applicable.
